# Enhancing the Conjugation of Nickel(II) Schiff Bases for High-Contrast Electrochromism

**DOI:** 10.3390/molecules31091433

**Published:** 2026-04-26

**Authors:** Jia-Xin Li, Li-Yi Zhang, Jin-Yun Wang, Feng-Rong Dai, Zhong-Ning Chen

**Affiliations:** 1 College of Chemistry and Materials Science, Fujian Normal University, Fuzhou 350007, China; lijiaxin@fjirsm.ac.cn; 2 State Key Laboratory of Structural Chemistry, Fujian Institute of Research on the Structure of Matter, Chinese Academy of Sciences, Fuzhou 350002, China; jy_wang@fjirsm.ac.cn; 3 Fujian College, University of Chinese Academy of Sciences, Fuzhou 350002, China

**Keywords:** electrochromism, nickel, metallopolymer, π-conjugation

## Abstract

In this work, we elaborately designed two nickel(II) Schiff base complexes (**NiL** and **NiL’**) with different π-conjugated systems (benzene vs. naphthalene) to prepare uniform metallopolymer films with nickel(II) chelates as repeating units on ITO substrates through oxidative electropolymerization. The π-conjugation extending from the benzene moiety to the naphthalene moiety greatly enhances the electron delocalization of the metallopolymer film, resulting in a significant increase in optical contrast from 25% (**[NiL]_n_**) to 80% (**[NiL’]_n_**). The solid-state electrochromic devices based on metallopolymer film **[NiL’]_n_** achieved a transmittance modulation of 71% and an electrochromic efficiency of 268.58 cm^2^ C^−1^. This work provides an effective strategy for developing low-cost and high-performance non-precious metal electrochromic materials through ligand conjugation engineering.

## 1. Introduction

Electrochromism (EC) refers to the optical properties of a compound or material, such as transmittance, absorption, and reflectance, undergoing reversible changes in the ultraviolet-visible-near-infrared (UV-Vis-NIR) regions under an external electric field [1,2,3,4,5], visually manifesting as significant reversible changes in color and transparency. Electrochromism exhibits broad application prospects in smart windows, antiglare rearview mirrors, low-power display screens, and wearable electronic devices [6,7,8,9]. The increasing global emphasis on environmental sustainability and the promotion of green technologies have led to a growing demand for high-performance, multifunctional electrochromic devices (ECDs) in the market. ECDs are composed of multi-layer films with a sandwich structure, typically including two transparent electrodes, an electrochromic layer, an ion-storage layer, and an electrolyte layer. The electrochromic layer is the core of electrochromic device [10,11,12] and is the dominant layer for optical modulation. Under an applied voltage, electrochemical redox reactions occur due to the gain and loss of electrons, accompanied by the insertion or removal of charge-balancing ions to maintain electrical neutrality, thereby causing changes in optical transmittance. Among numerous electrochromic materials, metal–organic complexes based on transition metals (such as Ru, Fe, Ni, Cu) have attracted widespread attention due to their well-defined and tunable molecular structures, rich redox states, and multicolor changes [13,14,15,16,17].

The design, synthesis, and preparation of high-quality functional films on conductive substrates are crucial for obtaining high-performance electrochromic devices. Researchers have developed various process approaches for preparing electrochromic films, such as spray coating, spin coating, vapor deposition, photothermal polymerization, and electrochemical polymerization [18,19,20,21,22,23,24,25,26]. Among them, electrochemical polymerization technology can directly grow conductive polymer films in situ on conductive substrates, and the film thickness is easily controllable by the amount of deposited charge. The prepared film is firmly bound to the substrate, providing a uniform electrochromic layer, which is an important direction promoting the development of this field [27,28,29].

Accurate control of the color contrast of electrochromic materials at the molecular level is the key to practical applications, in which adjusting the size of π-conjugated systems is an effective strategy [30,31,32,33]. Expanding the π-conjugation of ligands or the entire molecule can systematically reduce the HOMO-LUMO energy gap of the material, thereby modulating redox potentials, absorption properties, and coloring contrast. However, there are relatively few reports on the systematic study of the size effect of rigid conjugated moiety on the electrochromic behavior of complexes targeting specific metal centers, such as the abundant element nickel. Nickel complexes, as non-precious metal systems, have the advantages of low cost and environmental friendliness. Several nickel-based electrochromic systems have also been reported, such as poly[Ni(salen)] films [34] and Ni(II)-tripyridine metal polymers [14]. Recent studies have further explored nickel oxide-based films [35] and other nickel complexes [36] for electrochromic applications. However, the exploration of their electrochromic performance, especially the implementation of multi-color display based on structural design, still needs to be further explored. We envision that by constructing ligand frameworks with aromatic rings of different sizes (such as benzene and naphthalene moieties), high-contrast electrochromism is attainable while effectively regulating the π-conjugation and electron delocalization of the entire molecule through conjugated systems [37,38].

**Scheme 1:**
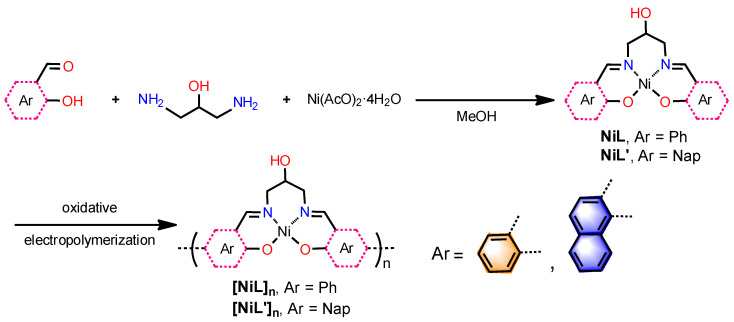
Synthetic route to metallopolymers **[NiL]_n_** and **[NiL’]_n_** through oxidative electropolymerization.

In this study, we focus on the electrochromic properties of the metallopolymer films derived from nickel(II) Schiff base complexes, with an emphasis on the effect of π-conjugation extension. We designed and synthesized two nickel(II) complexes, **NiL** [39,40] and **NiL’**, with similar structures but different π-conjugated systems (Scheme 1). This design strategy of modulating π-conjugation through rigid aromatic ring size is distinct from recent advances in electrochromic materials, such as the use of decoupled hybrid materials for high efficiency and capacity [41], the preparation of high-performance poly[Ni(salen)] films [34], or the exploration of magnetic properties in Ni-Mn complexes [39]. The metallopolymer films with excellent adhesion were prepared by electrochemical polymerization, and the differences in spectral electrochemical and dynamic electrochromic properties between the two metallopolymers were systematically compared. The conjugation extension from the benzene to the naphthalene moiety not only resulted in a cathodic shift in oxidation potential, but also significantly improved the optical modulation percentage, that is, the color change contrast increased from 25% to 80%. Furthermore, we fabricated an all-solid-state electrochromic device using **[NiL]_n_** and **[NiL’]_n_** as electrochromic layers, and compared and verified the high color rendering efficiency and good cycling stability of **[NiL’]_n_** in practical devices. In any case, this work provides an important experimental basis and theoretical reference for the development of low-cost and high color contrast nickel based electrochromic materials and devices through rational molecular design.

## 2. Results and Discussion

### 2.1. Synthesis and Characterization of Nickel(II) Complexes of Schiff Bases

**Figure 1:**
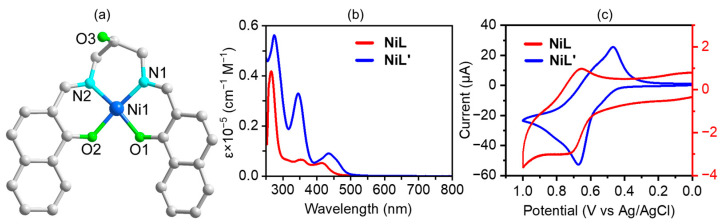
(**a**) A perspective view of **NiL’** from X-ray crystallography. The H atoms were omitted for clarity. (**b**) The UV-Vis absorption spectra of complexes **NiL** and **NiL’**. (**c**) The cyclic voltammograms of complexes **NiL** and **NiL’**. The red curve corresponds to the right vertical axis, and the blue curve corresponds to the left vertical axis.

As shown in Scheme 1, complexes **NiL** and **NiL’** were prepared by one-pot method through in situ Schiff base reaction using Ni^2+^ as a template. The compositions of complexes **NiL** and **NiL’** were confirmed by electrospray ionization mass spectrometry (ESI-MS) (Figures S1 and S2). Among them, **NiL’** crystals suitable for single crystal X-ray diffraction analysis were grown by slowly evaporating methanol solution (Figure 1a). Complex **NiL’** crystallizes in the *Pna*2_1_ space group, where the nickel (II) ion is chelated to tetradentate Schiff base L’. Ni(II) atom is bonded to two N and two O atoms, forming an approximate square-planar geometry. The *trans*-angles are 170.43 (16)° for N1-Ni1-O2 and 172.02 (15)° for N2-Ni1-O1. The *cis*-angles are 80.47 (9)°, 92.51 (12)°, 92.72 (11)°, and 94.71 (13)° for O2-Ni1-O1, N1-Ni1-O1, N2-Ni1-O2, and N2-Ni1-N1, respectively. The bond lengths between Ni1 and O1, O2, N1, N2 are 1.862 Å, 1.853 Å, 1.917 Å, and 1.852 Å, respectively.

Complexes **NiL** and **NiL’** exhibit two strong absorption peaks in the ultraviolet region, as well as a weak absorption peak in the visible light region below 500 nm. As shown in Figure 1b, the strong absorption peak in the range of 250–300 nm arises from the π→π* transition of the ligand. The absorption peak centered around 350 nm is primarily due to the n→π* transition within the ligand. The absorption peak at 400–500 nm is mainly attributed to the metal-to-ligand charge transfer (MLCT) transition. With the expansion of the π-conjugated system from L to L’, the energy gap of the π-π* orbitals is reduced, resulting in a decrease in transition energy and an overall redshift in the main absorption peak of complex **NiL’** relative to **NiL** (Figure S4). The UV-Vis absorption spectra showed no significant changes before and after irradiation with 365 nm ultraviolet light for 30 min, confirming the excellent photostability of the complexes **NiL** and **NiL’** (Figures S5 and S6).

**Figure 2:**
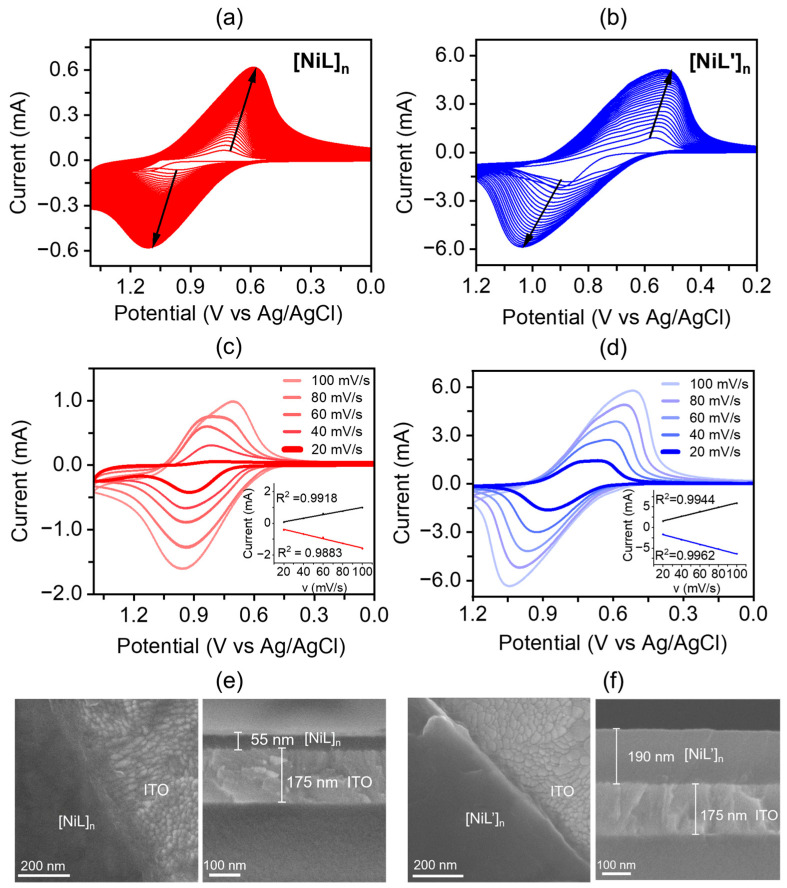
(**a**) The cyclic voltammograms of complex **NiL** in 0.1 M Bu_4_NClO_4_-MeCN solution at a scan rate of 0.1 V s^−1^ during the oxidative electropolymerization process. An ITO glass (2 × 2 cm^2^) and a Pt sheet (1 × 1 cm^2^) were used as the working electrode and the counter electrode with Ag/AgCl as the reference electrode. The arrows indicate the direction of increasing scan cycles. (**b**) The cyclic voltammograms of complex **NiL’** in 0.1 M Bu_4_NClO_4_-MeCN solution at a scan rate of 0.1 V s^−1^ during the oxidative electropolymerization process. An ITO glass (2 × 2 cm^2^) and a Pt sheet (1 × 1 cm^2^) were used as the working electrode and the counter electrode with Ag/AgCl as the reference electrode. The arrows indicate the direction of increasing scan cycles. (**c**) The cyclic voltammograms of metallopolymer film **[NiL]_n_**, recorded at different scan rates in a 0.1 M Bu_4_NClO_4_/MeCN solution. ITO glass (2 × 2 cm^2^) deposited with **[NiL]_n_** polymer film was used as the working electrode, Pt sheet (1 × 1 cm^2^) as the counter electrode, and Ag/AgCl as the reference electrode. Inset: showing a linear relationship between peak current and scan rate. (**d**) The cyclic voltammograms of metallopolymer film **[NiL’]_n_**, recorded at different scan rates in a 0.1 M Bu_4_NClO_4_/MeCN solution. ITO glass (2 × 2 cm^2^) deposited with **[NiL’]_n_** polymer film was used as the working electrode, Pt sheet (1 × 1 cm^2^) as the counter electrode, and Ag/AgCl as the reference electrode. Inset: showing a linear relationship between peak current and scan rate. (**e**) Surface and cross-sectional images of electrochromic film **[NiL]_n_** under scanning electron microscopy. (**f**) Surface and cross-sectional images of electrochromic film **[NiL’]_n_** under scanning electron microscopy.

The redox properties of complexes **NiL** and **NiL’** were investigated by cyclic voltammetry (CV) (Figure 2c). Within the range of 0 to 1.0 V (vs Ag/AgCl), both **NiL** (*E*_1/2_ = 0.69 V) and **NiL’** (*E*_1/2_ = 0.57 V) exhibit a quasi-reversible redox wave, respectively, which is attributed to the redox process of corresponding Schiff base ligand. Noticeably, the distinct cathodic potential shift in **NiL’** (*E*_1/2_ = 0.57 V) relative to **NiL** (*E*_1/2_ = 0.69 V) is largely ascribable to the larger π-conjugated system and better electron-rich character of naphthalene-containing L’ than that of benzene-containing L. The much smaller current intensity of **NiL** (the right vertical axis of Figure 1c) than that of **NiL’** (the left vertical axis of Figure 1c) is owing to the significantly worse solubility of the former. The frontier orbital energy levels of **NiL** and **NiL’** (Figure S4) reveal that the HOMO of **NiL’** rises from −5.60 eV to −5.45 eV compared to that of **NiL**, representing the increase of 0.15 eV. These results align well with the cathodic potential shift between **NiL** and **NiL’**. Furthermore, the HOMO-LUMO energy gap *E*_g_ of **NiL’** (3.72 eV) is reduced relative to that of **NiL** (3.81 eV), which accounts for the overall redshift observed in the absorption spectrum of **NiL’**.

### 2.2. Preparation and Characterization of Metallopolymer Films

Figure 2 shows the electrochemical polymerization process of complexes **NiL** (Figure 2a) and **NiL’** (Figure 2b). A three-electrode system with ITO glass as the working electrode, platinum plate as the counter electrode, and Ag/AgCl as the reference electrode was used for the oxidative electropolymerization of complexes **NiL** and **NiL’** on the surfaces of ITO using cyclic voltammetry at a scan rate of 0.1 V s^−1^. As electrochemical polymerization proceeded (Figure 2a,b), the area of the cyclic voltammogram gradually increased with the increase in the number of cycles, indicating that the **NiL** or **NiL’** monomers gradually polymerized and deposited on the ITO substrate. During the electropolymerization process of complex **NiL’**, not only is the increase in current intensity significantly faster than that of complex **NiL**, but also during the formation of metallopolymer film, the maximum current intensity of **[NiL’]_n_** (5.2 mA) was over eight times that of **[NiL]_n_** (0.62 mA). This can be attributed to the much higher solubility of complex **NiL’**, as well as the significant increase in the conjugated system of **NiL’**, which greatly facilitates electrochemical polymerization. The CV current intensity of ITO glass deposited with electropolymerized films **[NiL]_n_** (Figure 2c) and **[NiL’]_n_** (Figure 2d) gradually increases with the increase in scanning rate, indicating that the metallopolymer film is electroactive and bound to the conductive surface of ITO substrate. There is a linear correlation of peak current intensity with scan rate, indicating that it is controlled by surface restricted electron transfer kinetics. Scanning electron microscopy (SEM) (Figure 2e,f) showed that the metallopolymer formed a continuous and uniform film layer. Given that **NiL’** has better solubility and a more extended π-conjugated system is more facile for oxidative polymerization, the thickness of the metallopolymer film **[NiL’]_n_** (190 nm) is more than three times that of the film **[NiL]_n_** (55 nm). Atomic force microscopy (AFM) (Figures S7 and S8) studies have shown that the surface roughness of the film is smaller than 20 nm. X-ray photoelectron spectroscopy (XPS) confirmed that the oxidation state of nickel ions in the metallopolymer film is +2 (Figures S9 and S10). These experimental data demonstrate the successful preparation of structurally uniform and dense electrochromic films on ITO glass substrate through oxidative electropolymerization. To further verify the proposed polymer structure and coupling regiochemistry (Scheme 1), FT-IR analysis was performed (Figure S11). FT-IR analysis of the C–H out-of-plane deformation region (600–900 cm^−1^) provides evidence for polymer formation. Specifically, the disappearance of monomer peaks at ~793 and ~887 cm^−1^ and the emergence of a new band at 773 cm^−1^ indicate the formation of a polymer structure through C–C coupling during electropolymerization [42]. Additionally, natural population analysis (NPA) charge calculations indicate that the carbon atom at the position *para* to the oxygen atom carries a more positive charge than other ring positions, making it the most favorable site for electrophilic coupling (Figure S12). This *para*-coupling mechanism is consistent with literature precedents on poly[Ni(salen)] films [34] and the electrochemical dehydrogenative coupling of naphthalenes [43]. These results confirm the feasibility and superiority of the electrochemical polymerization approach for the preparation of electrochromic films of complexes **NiL** and **NiL’**.

### 2.3. Electrochromic Properties of Metallopolymer Films

**Figure 3:**
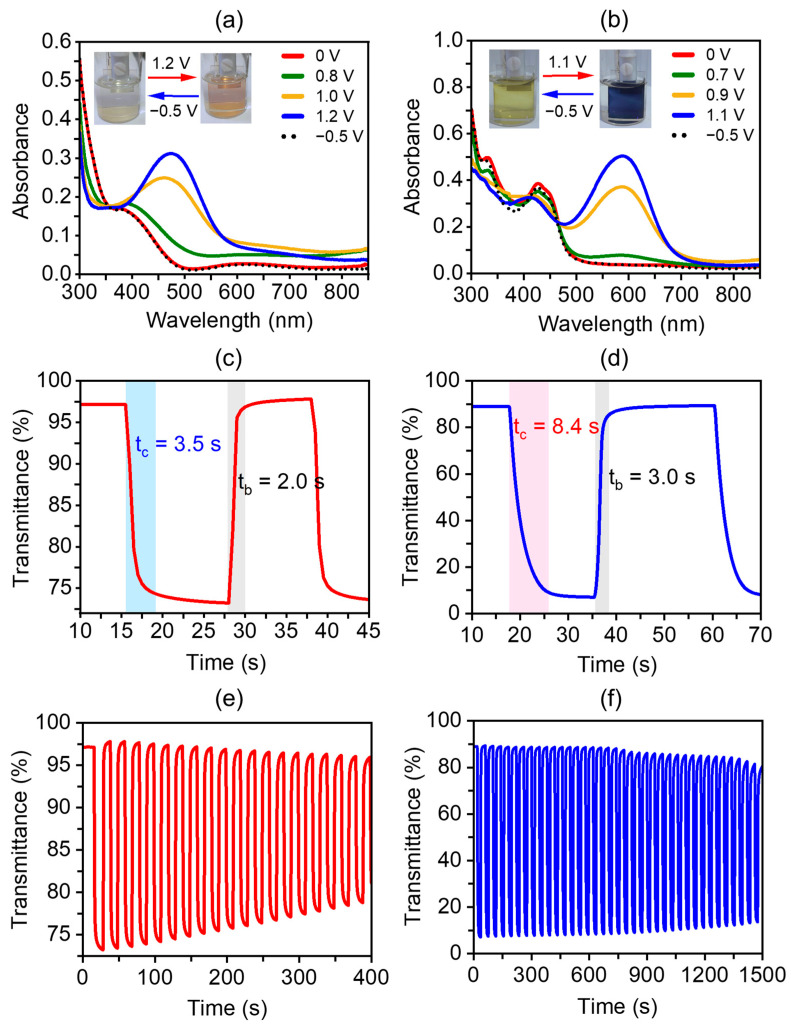
(**a**) The UV-Vis absorption spectra of electrochromic film **[NiL]_n_** on ITO glass in 0.1 M Bu_4_NClO_4_/MeCN solution at different applied voltages. The insets show the color change of the **[NiL]_n_** polymer film at different applied voltages. (**b**) The UV-Vis absorption spectra of electrochromic film **[NiL’]_n_** on ITO glass in 0.1 M Bu_4_NClO_4_/MeCN solution under different applied voltages. The insets show the color change of the **[NiL]_n_** polymer film at different applied voltages. (**c**) The electrochromic coloration and bleaching time of film **[NiL’]_n_** determined by monitoring the changes in transmission intensity at 475 nm. (**d**) The electrochromic coloration and bleaching time of film **[NiL’]_n_** determined by monitoring the changes in transmission intensity at 585 nm. (**e**) Electrochromic cycling stability of film **[NiL]_n_** at 475 nm. (**f**) Electrochromic cycling stability of film **[NiL’]_n_** at 585 nm.

Spectroelectrochemistry is useful for investigating the correlation between absorption spectra and electrochemical behaviors of electrochromic metallopolymer films as a function of applied potentials. To this end, we used a three-electrode system in a quartz colorimetric cuvette, combined with a UV-Vis absorption spectrometer and an electrochemical workstation, to characterize the electrochromic properties of **[NiL]_n_** and **[NiL’]_n_** films by spectral electrochemical combination. As shown in Figure 3a, **[NiL]_n_** film exhibits a new weak absorption peak at around 600 nm compared with the precursor monomer **NiL**. To explain the new absorption peak, we constructed a dimer molecular model considering the computational cost of the polymer. From the optimized structure (Figure S13, simulated by calculations), it is seen that the torsion angle between adjacent benzene rings after polymerization is 38.28°, which is small enough to allow for effective conjugation and cause a redshift in the absorption spectrum. The TD-DFT calculation results about the 600 nm absorption transition (Figure S14) indicate that both the hole and electron, representing the lower and upper levels of the transition, are primarily localized on the ligand **L** (90.23% for hole and 75.58% for the electron), thus resulting the enhancement of low-energy π-π* transitions in metallopolymer backbone. As the applied voltage continued to increase, a new absorption band appeared at 475 nm, and the absorption intensity reached its maximum at 1.2 V. The color of the film changed from almost colorless to orange red. These phenomena are attributable to the electrochemical oxidation of the metallopolymer under an applied voltage, which leads to a significant increase in the π-π* and n-π* transitions within the metallopolymer **[NiL]_n_**, as well as the MLCT transition as confirmed by the distributions of hole and electron corresponding to this absorption transition (Figure S15). It is worth noting that the color of the film **[NiL]_n_** can be reversibly restored to its original state when an external voltage of −0.5 V is applied.

In striking contrast to the film **[NiL]_n_**, the film **[NiL’]_n_** exhibits no significant absorption beyond 460 nm. Only absorption bands at 340 nm and 429 nm, similar to those of its precursor monomer, were observed, giving rise to a yellow-green appearance for the **[NiL’]_n_** (Figure 3b). This is attributed to the large torsion angle of 77.17° disrupting the conjugation between adjacent naphthalene rings after polymerization (Figure S13b), which essentially eliminates the charge transfer transitions between adjacent [**NiL’**] units in metallopolymer **[NiL’]_n_**. Thus, the absorption properties of metallopolymer **[NiL’]_n_** remain largely similar to those of the precursor monomer **NiL’**. With the increase in applied voltage, the film **[NiL’]_n_** underwent electrochemical oxidation, resulting in a slight decrease in the absorption intensity of the original bands. Meanwhile, a new absorption band appeared near 585 nm, attributed to the charge transfer transition within the metallopolymer upon electrochemical oxidation in the ligand **L’** with the contribution of 60.63% for hole and 96.69% for electron in the transition process (Figure S16). When the voltage increased to 1.1 V, the absorption band reached a steady state and the color of the film changed from yellow-green to deep blue. When the applied voltage dropped to −0.5 V, the deep blue reversibly returned to the original yellow-green.

In order to further investigate the cyclic stability of the electrochromic process of metallopolymer films, the switching ability of electrochromic films was studied using dual potential step chronoamperometry (Figure 3c,d). After applying a voltage of 1.1 V, the film **[NiL]_n_** rapidly converted from its initial state (almost colorless) to an oxidized state (orange-red), with a coloring time (t_c_) of 3.5 s. Under an applied voltage of −0.5 V, the film reversibly returned to its initial state, with a bleaching time (t_b_) of 2.0 s (Figure 3c). The optical transmittance change (∆T) in coloring/bleaching processes is 25%. The film **[NiL]_n_** exhibits relative stability over 20 switching cycles, with optical contrast decreasing to 18% (Figure 3e). In sharp contrast, the film **[NiL’]_n_** rapidly converted from its initial state (yellow green) to an oxidized state (deep blue) after applying a voltage of 1.1 V, with a coloring time (t_c_) of 8.4 s. Under an applied voltage of −0.5 V, it reversibly returned to its initial state, with a bleaching time (t_b_) of 3.0 s (Figure 3d). The coloring/bleaching optical transmittance change (∆T) is 80%. The film **[NiL’]_n_** exhibits relative stability during 38 switching cycles, with an optical contrast decrease to 70% (Figure 3f). From this, it can be seen that both films **[NiL]_n_** and **[NiL’]_n_** exhibit good coloring and bleaching response. Extending the π-conjugation from the benzene to the naphthalene moiety narrows the HOMO–LUMO gap (from 3.81 eV to 3.72 eV, Figure S4) and enhances metal-to-ligand charge transfer (MLCT) efficiency, which directly promotes a larger optical modulation (from 25% to 80%, Figure 3c,d). Moreover, the improved electron delocalization in **[NiL’]_n_** lowers the reorganization energy for charge transfer, resulting in a cathodic shift in oxidation potential (from 0.69 V to 0.57 V, Figure 1c). Consequently, the conjugation regulation by replacing benzene moiety to naphthalene moiety results in film **[NiL’]_n_** having a much higher electrochromic optical contrast than that of **[NiL]_n_**. The higher optical contrast makes the film **[NiL’]_n_** a more promising and efficient electrochromic material.

### 2.4. Solid-State Electrochromic Devices

**Figure 4:**
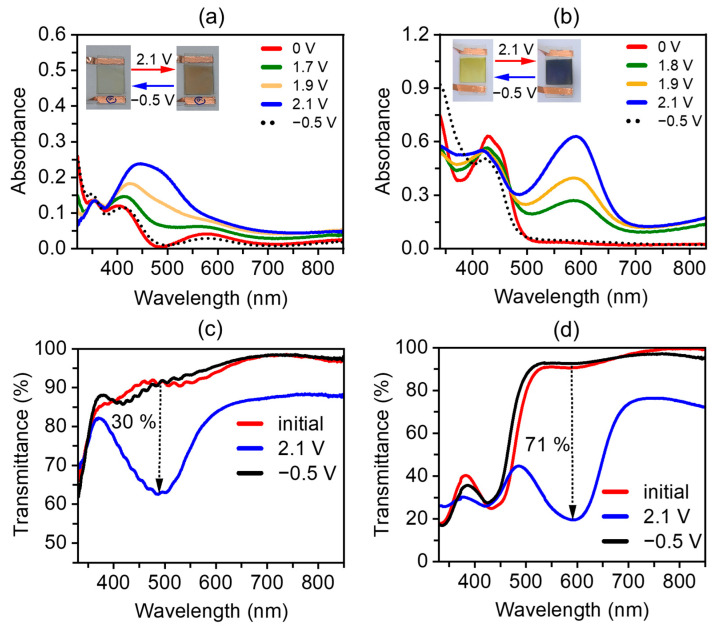
(**a**) Spectroelectrochemical characteristics of **[NiL]_n_**/TiO_2_ electrochromic devices in the voltage range of −0.5 V to 2.1 V. The insets show the color change of the electrochromic devices at different applied voltages. (**b**) Spectroelectrochemical characteristics of **[NiL’]_n_**/TiO_2_ electrochromic devices in the voltage range of −0.5 V to 2.1 V. The insets show the color change of the electrochromic devices at different applied voltages. (**c**) Transmission spectra of **[NiL]_n_**/TiO_2_ electrochromic devices at different voltages. (**d**) Transmission spectra of **[NiL’]_n_**/TiO_2_ electrochromic devices at different voltages.

In order to further evaluate the electrochromic properties of metallopolymer films **[NiL]_n_** and **[NiL’]_n_**, a systematic exploration was conducted by fabricating solid-state electrochromic devices. A sandwich structure device with ITO/electrochromic layer/photocuring electrolyte/TiO_2_/ITO was prepared using metallopolymer films **[NiL]_n_** or **[NiL’]_n_** as electrochromic layers, TiO_2_ as ion storage layer, and photocurable electrolyte as an ion transport layer. As shown in Figure 4a, akin to the absorption spectrum of the electrochromic film, the device based on **[NiL]_n_**/TiO_2_ exhibits a weak absorption peak at 600 nm in the visible region, attributed to the low-energy π-π* transition and MLCT transition of the metallopolymer backbone. The initial state of the device is almost colorless. As the applied voltage increased by 2.1 V, the **[NiL]_n_**/TiO_2_ device changed to orange red, while a new absorption peak appeared at 475 nm. The contrast of the optical transmittance of coloring/bleaching reached 30% (Figure 4c). By applying a voltage of −0.5 V, the device can be fully restored to its original state.

In striking contrast to **[NiL]_n_**/TiO_2_, **[NiL’]_n_**/TiO_2_ electrochromic devices exhibited yellow-green in their original state. As the applied voltage increased, the electrochromic layer underwent electrochemical oxidation, and the intensity of the absorption peak centered at 429 nm slightly weakened, accompanied by the appearance of a new strong absorption band near 585 nm. When the voltage increased to 2.1 V, the device changed to deep blue, and the color change was significant and uniform (Figure 4b), with an optical transmittance change of 71% (Figure 4d). Undoubtedly, by replacing the benzene ring with a naphthalene ring to expand the π-conjugated system, the coloring/bleaching contrast of electrochromic devices dramatically increased from 30% (**[NiL]_n_**/TiO_2_) to 71% (**[NiL’]_n_**/TiO_2_).

**Figure 5:**
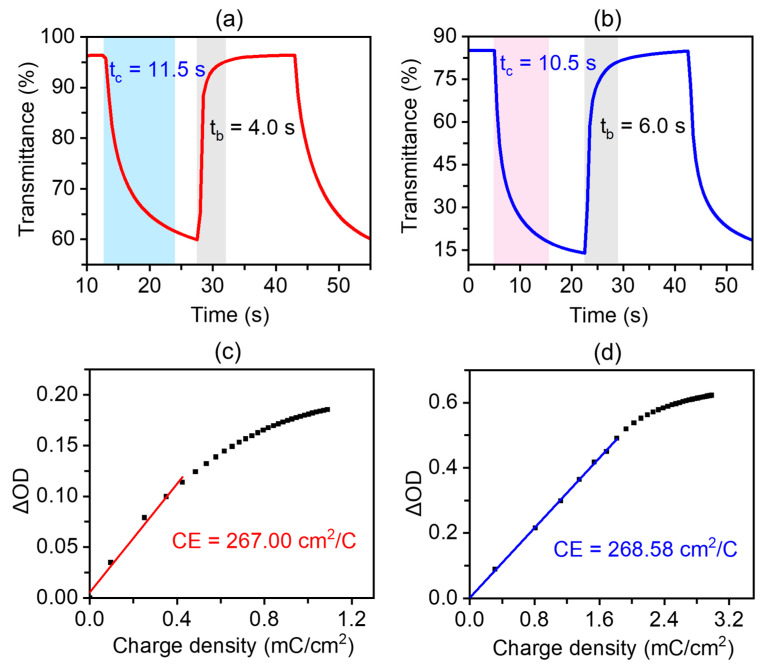
(**a**) Electrochromic response time of **[NiL]_n_**/TiO_2_ electrochromic device determined by monitoring the changes in transmission intensity at 475 nm. (**b**) The electrochromic response time of **[NiL’]_n_**/TiO_2_ electrochromic devices determined by monitoring the changes in transmission intensity at 585 nm. (**c**) The coloring efficiency of **[NiL]_n_**/TiO_2_ electrochromic devices at 475 nm. (**d**) The coloring efficiency of **[NiL’]_n_**/TiO_2_ electrochromic devices at 585 nm.

The response time of electrochromic devices was evaluated using the dual potential step current method, with step potentials ranging from −0.5 to 2.1 V applied. The coloring time (t_c_) and bleaching time (t_b_) of the **[NiL]_n_**/TiO_2_ electrochromic device during the electrochromic process are 11.5 s and 4.0 s, respectively, based on a 95% change in transmittance (Figure 5a). The calculation formula for electrochromic coloring efficiency (CE) is *η* = ∆OD/∆Q = log (t_b_/t_c_)/∆Q, where ∆OD is the change in optical density and ∆Q is the injected charge. The coloring efficiency (CE) measured at 475 nm is 267.00 cm^2^ C^−1^ (Figure 5c) and the bleaching efficiency (BE) is 319.69 cm^2^ C^−1^ (Figure S17). In contrast, the **[NiL’]_n_**/TiO_2_ electrochromic device has a coloring time (t_c_) of 10.5 s and a bleaching time (t_b_) of 6.0 s (Figure 5b). The CE measured at 585 nm was 268.58 cm^2^ C^−1^ (Figure 5d) and the BE was 337.59 cm^2^ C^−1^ (Figure S18).

**Figure 6:**
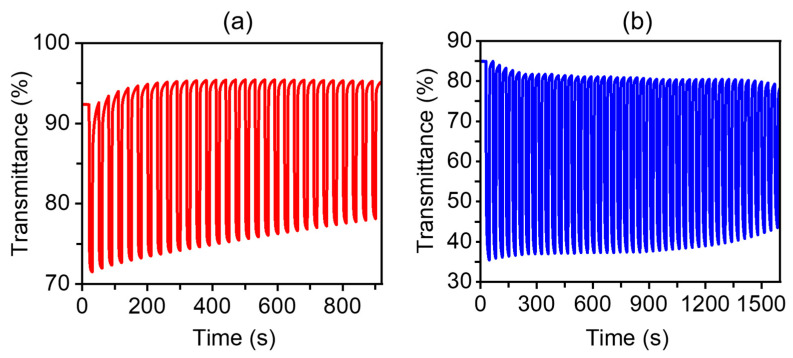
(**a**) Cycling stability of **[NiL]_n_**/TiO_2_ electrochromic device monitored at 475 nm at applied voltages of −0.5 V and 2.0 V. (**b**) Cycling stability of **[NiL’]_n_**/TiO_2_ electrochromic device monitored at 475 nm at applied voltages of −0.5 V and 2.0 V.

The cycling stability of the solid-state electrochromic devices was further evaluated by double-step potential chronoamperometry between −0.5 V and 2.0 V. The maximum transmittance difference (ΔT) between the original (reduced) and oxidized states of the **[NiL]_n_**/TiO_2_ electrochromic device at 475 nm is 22% (Figure 6a). After 30 switching cycles between −0.5 V and 2.0 V, it was observed that the optical contrast remained at 80% of the initial value. The electrochromic device of **[NiL’]_n_**/TiO_2_ has an optical contrast (∆T) of about 50% at a wavelength of 585 nm (Figure 6b), and after 48 cycles, the optical contrast remained at 80% of the initial value. Anyway, all experimental results demonstrated that the larger conjugated system with a naphthalene ring in place of a benzene ring dramatically improves the cycling stability and electrochromic contrast of metallopolymer film.

## 3. Materials and Methods

### 3.1. General Information

All of the manipulations were performed under a dry nitrogen atmosphere by using Schlenk techniques. Solvents were dried by standard methods and distilled prior to use except for those of spectroscopic grade for the spectroscopic measurements. Unless otherwise noted, starting materials were obtained from commercial suppliers and used without further purification. Indium tin oxide (ITO)-coated glass slides (20 × 30 × 1 mm, sheet resistance *R*_s_ = 6 Ω/sq) were purchased from South China Xiangcheng Technology Co., Ltd (Shenzhen, China). Prior to use, the ITO slides were ultrasonically cleaned sequentially with alkaline detergent, deionized water, ethanol, acetone, and isopropanol.

The ^1^H NMR spectral measurements were performed on a Bruker AVANCE III 400 MHz instrument (Bruker Corporation, Billerica, MA, USA) and used trimethylsilane (TMS) as the internal standard. The UV-Vis absorption spectra were measured on a Shimadzu UV-2600i UV-Vis spectrophotometer (Shimadzu, Suzhou, China). The electrospray ionization mass spectrometry (ESI-MS) was recorded on an Agilent 6230 mass spectrometer (Agilent Technologies, Santa Clara, CA, USA) using a dichloromethane-methanol mixture in mobile phases. The topology of electrochromic film was investigated with scanning electron microscopy (SEM) using JEOL6700F (JEOL Ltd., Tokyo, Japan), and atomic force microscopy (AFM) using Dimension Icon (Bruker Corporation, MA, USA). Electrochemical experiments were performed on a CHI660E electrochemical workstation (Chenhua, Shanghai, China) at room temperature unless otherwise mentioned.

### 3.2. Synthesis of Complex **NiL**

Instead of the stepwise procedure reported in the literature [40], which involved the isolation of the free Schiff base ligand, **NiL** was synthesized by a modified one-pot method. Under anhydrous and anaerobic conditions, 1,3-diamino-2-hydroxypropane (0.55 g, 6.14 mmol) was added to a methanol solution (50 mL) of salicylaldehyde (1.50 g, 12.3 mmol). After stirring for 2 h, nickel(II) acetate was added to the reaction mixture, and stirring was continued for another 2 h, yielding a green suspension. The precipitate was collected by vacuum filtration to afford the pale green target product **NiL** (1.90 g, 88%). The complex exhibited insufficient solubility in any solvent to obtain a ^1^H NMR spectrum. HR-MS (*m*/*z*): calcd for [M+H]^+^: 355.0587; found: 355.0628.

### 3.3. Synthesis of Complex **NiL’**

Under anhydrous and anaerobic conditions, 1,3-diamino-2-hydroxypropane (0.39 g, 4.35 mmol) was added to a methanol solution (50 mL) of 1-hydroxynaphthalene-2-carboxaldehyde (1.5 g, 8.7 mmol). After stirring for 2 h, nickel(II) acetate was added to the reaction mixture, and stirring was continued for another 2 h, giving a green suspension. The precipitate was collected by vacuum filtration to yield a yellow-green target product **NiL’** (1.7 g, 86%). ^1^H NMR (400 MHz, CDCl_3_) *δ* (ppm) = 8.54 (d, *J* = 8.0 Hz, 2H), 7.70–7.40 (m, 6H), 7.21 (s, 2H), 6.94 (dd, *J* = 12.0, 8.0 Hz, 4H), 4.20 (s, 1H), 3.88 (d, *J* = 24.0 Hz, 2H), 3.40 (d, *J* = 12.0 Hz, 2H) (Figure S3). HR-MS (*m*/*z*): calcd for [M+H]^+^: 455.0900; found: 455.0924.

### 3.4. X-Ray Crystallography

X-ray single-crystal diffraction data were collected on a Bruker D8 Venture diffractometer using IμS 3.0 microfocus source Mo-Kα radiation (*λ* = 0.71073 Å) and PHOTON II CPAD detector (Bruker AXS GmbH, Karlsruhe, Germany). Frames were integrated with the Bruker SAINT software package (V8.38A) using a SAINT algorithm. Data were corrected for absorption effects using the multi-scan method (SADABS) [44]. The structure was solved and refined using the Bruker SHELXTL Software Package (Version 2014), a computer program for automatic solutions of crystal structures and refined by the full-matrix least-squares method with ShelXle Version 4.8.6, a Qt graphical user interface for the SHELXL [45]. CCDC 2525270 contains the supplementary crystallographic data for this paper. These data can be obtained free of charge from the Cambridge Crystallographic Data Centre via www.ccdc.cam.ac.uk/data_request/cif (accessed on 26 January 2026).

### 3.5. Preparation of Metallopolymer Films

The metallopolymer films (**[NiL]_n_** and **[NiL’]_n_**) were fabricated via electropolymerization in a standard three-electrode system, employing an ITO glass (2 × 2 cm^2^) as the working electrode, an Ag/AgCl as the reference electrode, and a platinum sheet (1 × 1 cm^2^) as the counter electrode. The electropolymerization was performed by cyclic voltammetry with the scan potential between 0.2 V and 1.4 V at a scan rate of 0.1 V/s in a 20 mL MeCN solution of 0.1 M Bu_4_NClO_4_ and 0.1 M **NiL** or **NiL’**. The electropolymerized film was thoroughly rinsed with absolute ethanol to remove residual electrolyte and impurities and dried in the air.

### 3.6. Characterization of **[NiL]_n_** and **[NiL’]_n_** Film

The in situ synchronizing spectroelectrochemistry studies were conducted by the combination of CHI 660E electrochemical workstation and UV-2600i UV-Vis spectrophotometer (Shimadzu, Suzhou, China). The **[NiL]_n_** or **[NiL’]_n_** film was used as working electrode to set up the three-electrode system in a quartz using platinum mesh (0.5 × 0.5 cm^2^) as the counter electrode and Ag/AgCl as the reference electrode. The cuvette was placed in the sample holder of UV-Vis spectrometer and connected to the electrochemical workstation. The absorption and transmittance spectra of films under different applied voltages were collected simultaneously during the electrochemical measurements.

### 3.7. Fabrication of Solid-State Electrochromic Device

The electrochromic devices (ECDs) were fabricated with a sandwich structure of ITO/electrochromic film/solid-state electrolyte/TiO_2_/ITO. The TiO_2_ was chosen as the ion storage layer and deposited on the ITO glass through the pulling technology and annealed at 400 °C for 10 min. The UV-curable electrolyte was prepared by mixing 0.2 g of 2-hydroxyethyl acrylate and 0.4 g of pentaerythritol triacrylate in 1.5 mL of a 0.5 M LiClO_4_ propylene carbonate (PC) solution, followed by the addition of 3 wt% of diphenyl(2,4,6-trimethylbenzoyl)phosphine oxide (TPO) photoinitiator. Then 18 µL of the prepared UV-curable electrolyte was deposited onto TiO_2_-coated ITO glass. Subsequently, the ITO glass deposited with electrochromic film was then carefully aligned to cover TiO_2_-coated ITO glass, forming a sandwich structure. The entire assembly was subsequently cured under UV (365 nm) irradiation for 60 s to finalize the solid-state device, which was used for subsequent characterizations.

### 3.8. Characterization of Solid-State Electrochromic Device

The device was directly placed in the sample holder of UV-vis-NIR spectrometer and connected to the electrochemical analyzer using a two-electrode configuration. The absorption or transmittance spectra of devices under different applied voltages were monitored simultaneously during the electrochemical measurements. The electrochromic performance of the device was evaluated by the optical contrast, switching time, and coloration efficiency.

### 3.9. Computational Method

The theoretical calculations were implemented by using Gaussian 16 program package [46]. The geometrical structures of **NiL**, **NiL’**, **[NiL]_2_**, **[NiL’]_2_**, **[NiL]^2+^**, and **[NiL’]^2+^** molecules in the ground states were respectively optimized by a density functional theory (DFT) method with the hybrid functional of M06 [47]. Then, in order to analyze the spectroscopic properties, 60 singlet excited-states were calculated, respectively, based on the optimized structures in the ground states to determine the vertical excitation energies by time-dependent density functional theory (TD-DFT) [48,49] with the same functional theory used in the optimization process. In these calculations, the Land2TZ(f) basis set and the effective core potentials (ECPs) were used to describe the Ni atoms [50,51], while other non-metal atoms of N, O, C, and H were described by the all-electron basis set of 6-311G**. Visualization of the HOMO and LUMO orbitals, hole and electron (isovalue = 0.0004) [52] plots in the absorption transition processes were performed by GaussView 6.0.16.

## 4. Conclusions

We demonstrate a simple and effective strategy for preparing high-performance electrochromic devices based on oxidative electropolymerization to provide high-quality metallopolymer films. The obtained metallopolymer films **[NiL]_n_** and **[NiL’]_n_** were systematically characterized by cyclic voltammetry, UV-Vis spectroscopy, SEM, and AFM. Replacing benzene moiety with naphthalene having larger π-conjugation gives rise to dramatically improved electrochromic properties such as better electrochemical cycle stability and higher electrochromic contrast. The solid-state electrochromic devices fabricated with metallopolymer films **[NiL’]_n_** achieve a 71% transmittance modulation, superior coloring efficiency, and excellent cycling stability. Our research indicates that through rational molecular design, low-cost and high-performance nickel based electrochromic materials and devices provide an important experimental basis and theoretical reference for the development of new electrochromic technologies in the future.

## Data Availability

Dataset available upon request from the authors.

## Supplementary Materials

The following supporting information can be downloaded at: https://www.mdpi.com/article/10.3390/molecules31091433/s1, **Table S1**. Crystallographic Data for **NiL’**; **Table S2.** Comparison of ECDs Performance of Recently Reported EC Materials and Our Nickel(II)-based EC Film via Electropolymerization [53,54,55,56,57,58,59,60,61,62,63,64,65,66]; **Figure S1**. ESI-MS spectrum of **NiL**; **Figure S2**. ESI-MS spectrum of **NiL’**; **Figure S3**. The ^1^H NMR spectrum of **NiL’**; **Figure S4**. Contour plots (isovalue = 0.02) of HOMO and LUMO orbitals with energy level diagram of **NiL** and **NiL’** by M06 functional; **Figure S5**. The UV-Vis spectra of **NiL** before and after irradiation with 365 nm ultraviolet light for 30 minutes; **Figure S6**. The UV-Vis spectra of **NiL’** before and after irradiation with 365 nm ultraviolet light for 30 minutes; **Figure S7**. AFM image of [**NiL]_n_** on ITO; **Figure S8**. AFM image of [**NiL’]_n_** on ITO; **Figure S9**. The XPS spectra of [**NiL]_n_** metallopolymer film; **Figure S10**. The XPS spectra of [**NiL’]_n_** metallopolymer film; **Figure S11**. The FT-IR spectra of **NiL’** monomer and [**NiL’]_n_** metallopolymer film; **Figure S12**. The natural population analysis (NPA) charge of **NiL** and **NiL’**. **Figure S13**. The optimized structure of the dimer (a) **[NiL]_2_**, and (b) **[NiL’]_2_**, indicating a smaller angle between the two benzene rings in **[NiL]_2_**; **Figure S14**. Contour plots of hole and electron (isovalue = 0.0004) in the transition process of ~600 nm and the contribution of molecular fragment (%) for **[NiL]_2_**; **Figure S15**. Contour plots of hole and electron (isovalue = 0.0004) in the transition process of 475 nm and the contribution of molecular fragment (%) for **[NiL]^2+^**; **Figure S16**. Contour plots of hole and electron (isovalue = 0.0004) in the transition process of 585 nm and the contribution of molecular fragment (%) for **[NiL’]^2+^**; **Figure S17**. Plot of the optical density (ΔOD) at 475 nm versus the charge density for the **[NiL]_n_**/TiO_2_ electrochromic device during the bleaching process (after applying a potential of 2.1 V) under an applied potential of −0.5 V; **Figure S18**. Plot of the optical density (ΔOD) at 585 nm versus the charge density for the **[NiL’]_n_**/TiO_2_ electrochromic device during the bleaching process (after applying a potential of 2.1 V) under an applied potential of −0.5 V.

## Abbreviations

The following abbreviations are used in this manuscript:

EC	electrochromism
ECD	electrochromic device
HOMO	highest occupied molecular orbital
LUMO	lowest unoccupied molecular orbital
MLCT	metal-to-ligand charge transfer
ITO	indium tin oxide
CV	cyclic voltammetry
CE	coloring efficiency
BE	bleaching efficiency
