# Supplementary material for: Enhancing the Conjugation of Nickel(II) Schiff Bases for High-Contrast Electrochromism
Source: Molecules. 2026 Apr 26;31(9):1433. doi: 10.3390/molecules31091433 (PMC13165144; doi:10.3390/molecules31091433)
Supplement: Supplementary file 1 [file molecules-31-01433-s001.zip › molecules-4257613-supplementary.pdf]

Supporting Information

Enhancing the Conjugation of Nickel(II) Schiff Bases for High-Contrast Electrochromism

Jia-Xin Li,[1,2] Li-Yi Zhang,[2,*] Jin-Yun Wang,[2] Feng-Rong Dai,[2,*] Zhong-Ning Chen[2,*]

[1] *College of Chemistry and Materials Science, Fujian Normal University, Fuzhou 350007, China*

[2] *State Key Laboratory of Structural Chemistry, Fujian Institute of Research on the Structure of Matter, Chinese Academy of Sciences, Fuzhou, Fujian 350002, China*

[3] *Fujian College, University of Chinese Academy of Sciences, Fuzhou 350002, China*

* *Correspondence: zhangliyi@fjirsm.ac.cn (L.-Y.Z.); dfr@fjirsm.ac.cn (F.-R.D.); czn@fjirsm.ac.cn (Z.-N.C.)*

Table S1. Crystallographic Data for **NiL'**.

	**NiL'**
Empirical formula	$C_{25}H_{20}N_2NiO_3$
Formula weight	455.142
Temperature (K)	230(2)
Wavelength	0.71073
Crystal system	Orthorhombic
Space group	Pna2(1)
a (Å)	20.5184(19)
b (Å)	13.8271(12)
c (Å)	7.3371(5)
α (°)	90
β (°)	90
γ (°)	90
V (Å^3)	2081.6(3)
Z	4
D (calcd) (g cm^{-3})	1.452
μ (Mo K_α) (mm^{-1})	0.962
F(000)	944
θ range (°)	3.296−25.369
Reflections collected / unique	12025 / 3559 [R_{int} = 0.0543]
Data / restraints / parameters	3559 / 1 / 282
GOF	1.024
R_1 (I>2σ(I))	0.0471
wR_2 (I>2σ(I))	0.1185
R_1 (all data)	0.0518
wR_2 (all data)	0.1220

Table S2. Comparison of ECDs Performance of Recently Reported EC Materials and Our Nickel(II)-based EC Film via Electropolymerization.

EC materials	ΔT (%)	t_c (s)/t_b (s)	CE (cm^2/C)	Cyclic stability	Ref.
WO_3	42.1 (550 nm)	6.8/5.9	98.5	97.4% after 2000 cycles	[53]
WO_3	41.0 (550 nm)	62.5/33.4	97.9	52.6% after 500 cycles	[54]
WO3/NiO	50.8 (633 nm)	4.0/0.8	86.6	77.4% after 15000 cycles	[55]
PEDOT:PSS	35 (548 nm)	25/25	-	81% after 1000 cycles	[56]
PEDOT:PSS	32 (650 nm)	0.25/0.52	237.62	50	[57]
H-PEDOT	35.1 (490 nm)	0.9/0.8	227	51%	[58]
SiO_2/PEDOT	27.3 (490 nm)	1.1/1.7	179	50% after 1000 cycles	[58]
HPV^{2+}/HQ	82.9 (607 nm)	~180	243	>10 cycles	[59]
Prussian blue	63 (652 nm)	2.6/3.5	131.5	82% after 1000 cycles	[60]
PUNZ	53.3 (606nm)	1.9/4.3		95% after 50 cycles	[61]
$[MnL]_n$	40 (490 nm)	1.4/0.4	337.5	80% after 10000 cycles	[62]
$[MnL]_n$	73 (740 nm)	2.2/0.4	397.1	80% after 125 cycles	[62]
Nb_2O_5	40 (660 nm)	10/6	-	20	[63]
V_2O_5	25 (633 nm)	6.3/11.8	-	-	[64]
P3	46 (510 nm)	0.9/1.1	415	100 cycles	[65]
POV	16 (550 nm)	11.7/5.1	34.9	71% after 200 cycles	[66]
$[NiL]_n$	22 (475 nm)	11.5/4	267.00	80% after 30 cycles	This work
$[NiL']_n$	50 (585 nm)	10.5/6	268.58	80% after 48 cycles	

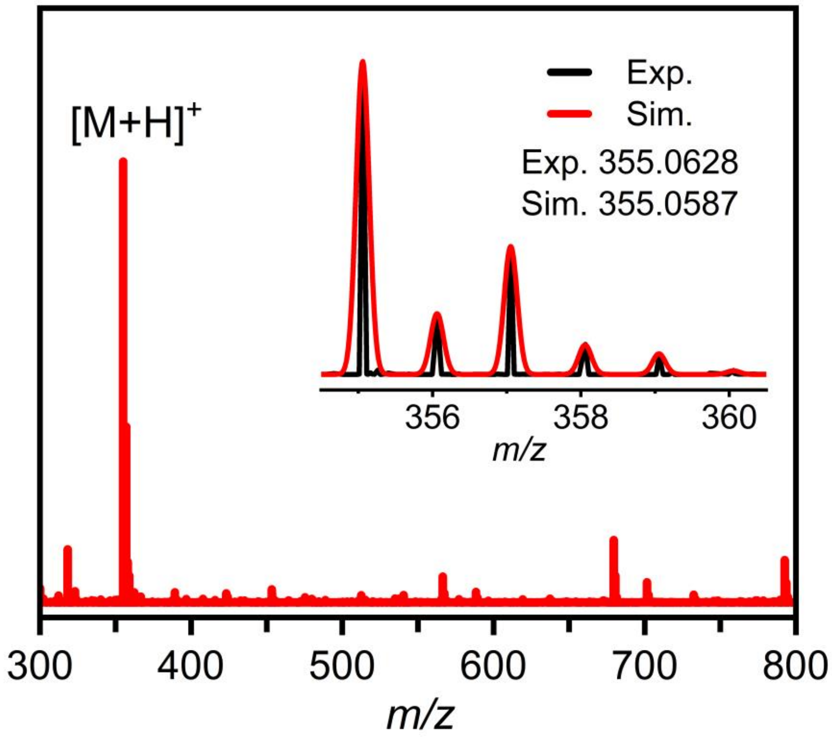

Figure S1. ESI-MS spectrum of **NiL**.

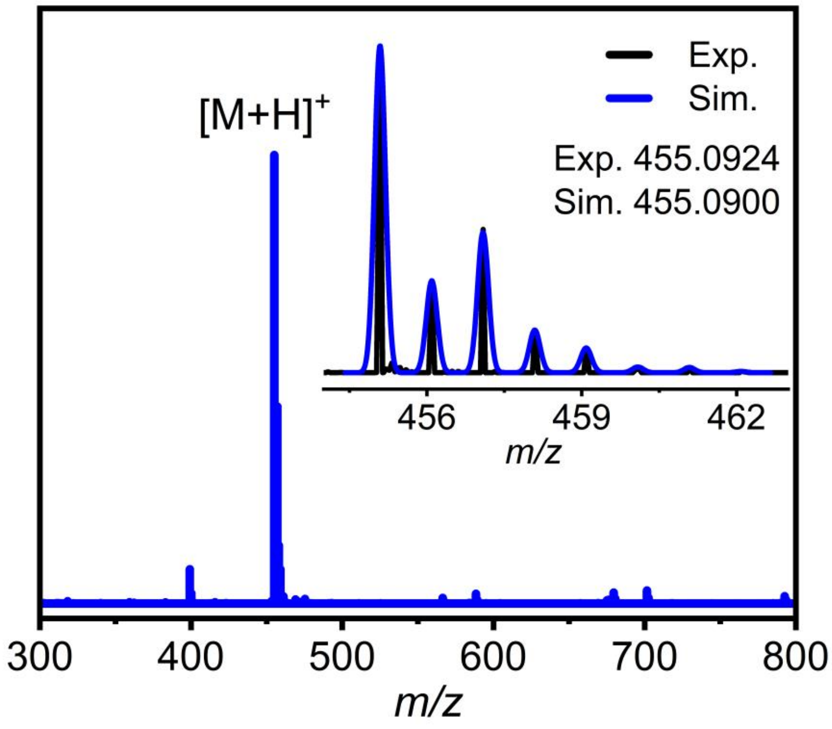

Figure S2. ESI-MS spectrum of **NiL'**.

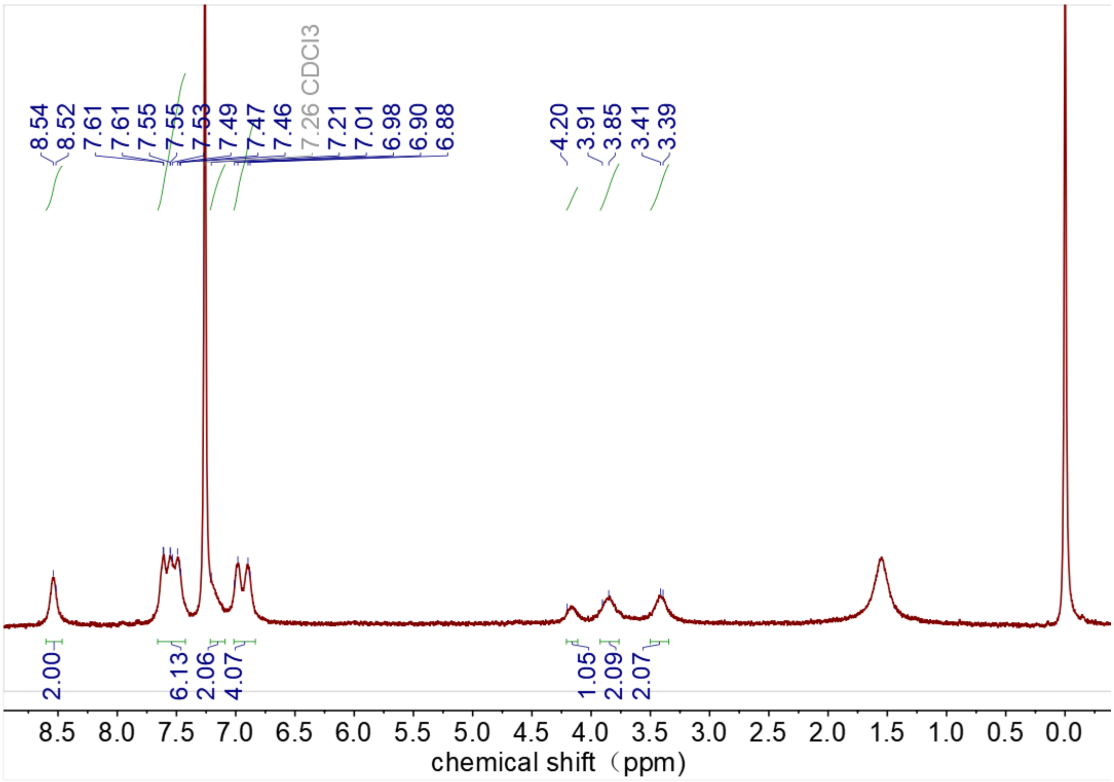

Figure S3. The ^{1}H NMR spectrum of **NiL'**.

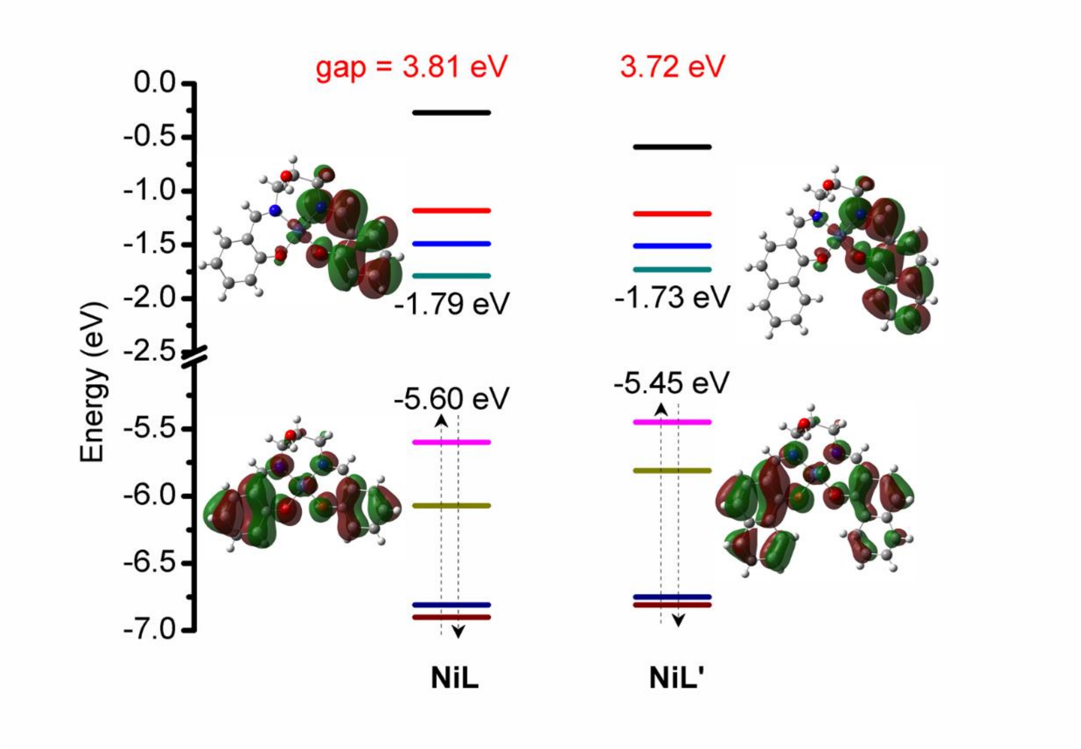

Figure S4. Contour plots (isovalue = 0.02) of HOMO and LUMO orbitals with energy level diagram of **NiL** and **NiL'** by M06 functional.

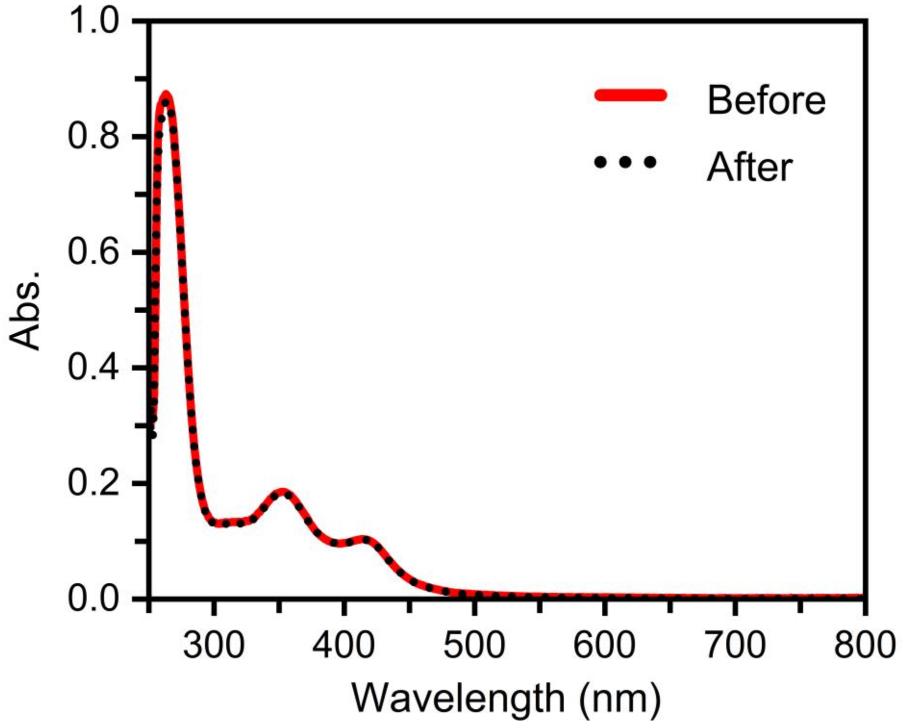

Figure S5. The UV-Vis spectra of **NiL** before and after irradiation with 365 nm ultraviolet light for 30 minutes.

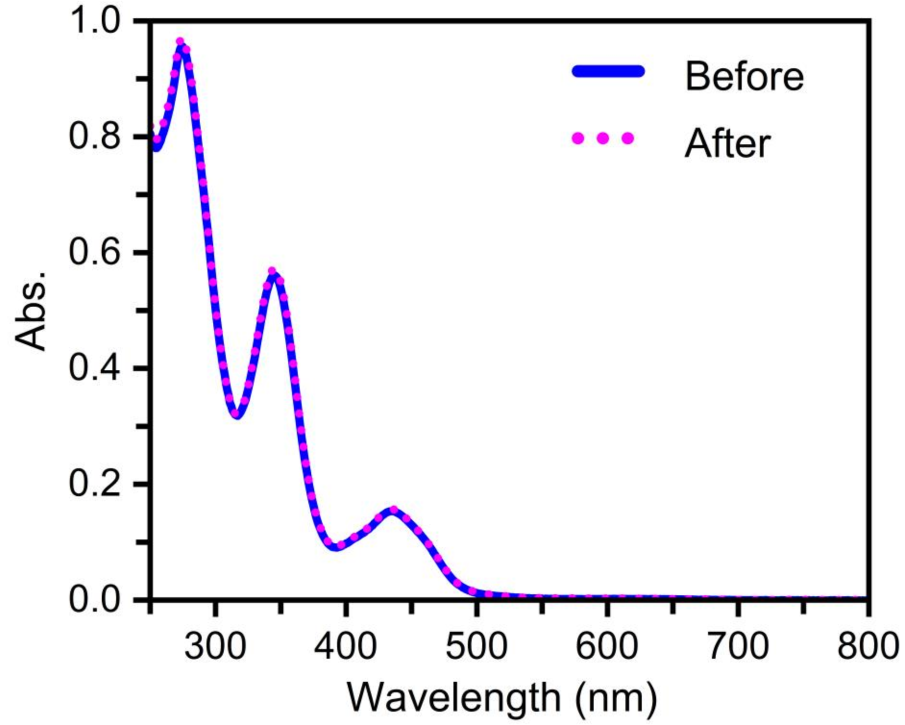

Figure S6. The UV-Vis spectra of **NiL'** before and after irradiation with 365 nm ultraviolet light for 30 minutes.

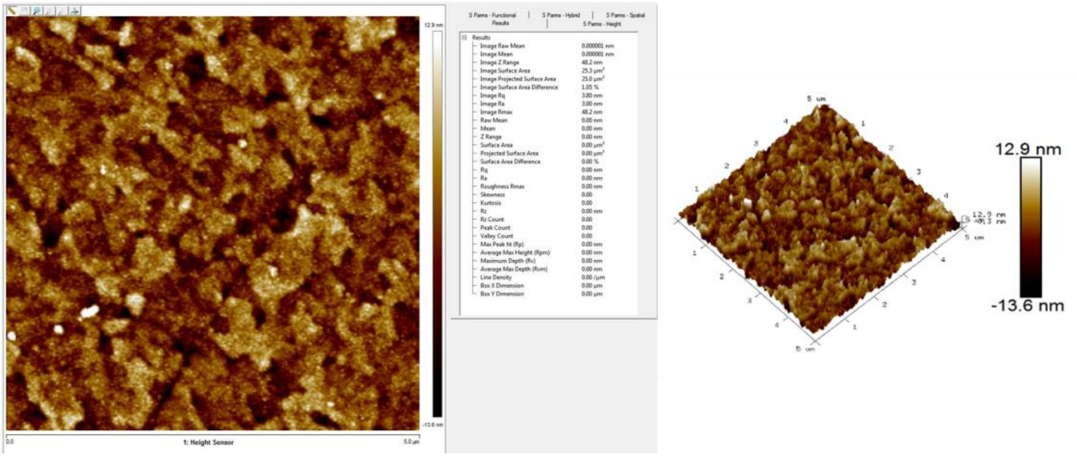

Figure S7. AFM image of [**NiL**]$_n$ on ITO.

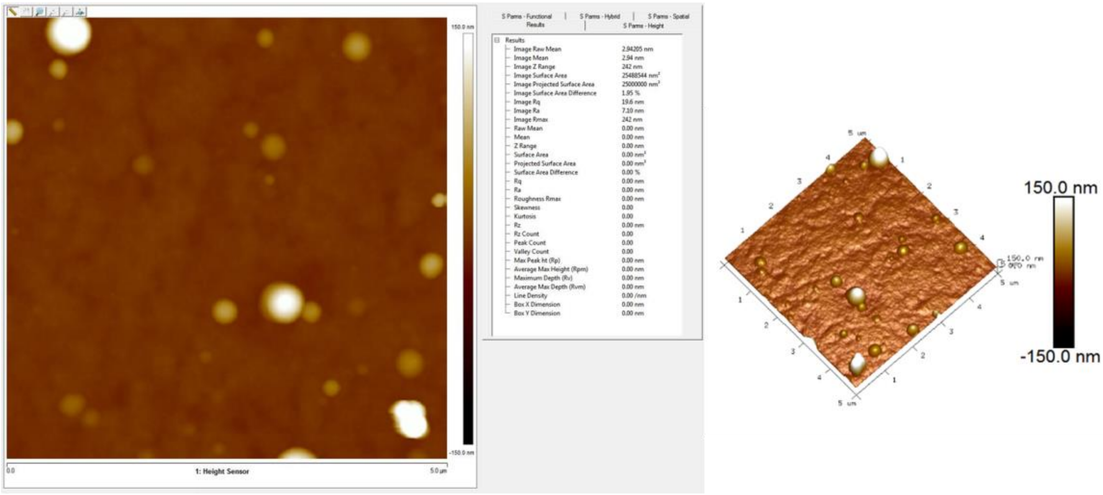

Figure S8. AFM image of [**NiL'**]$_n$ on ITO.

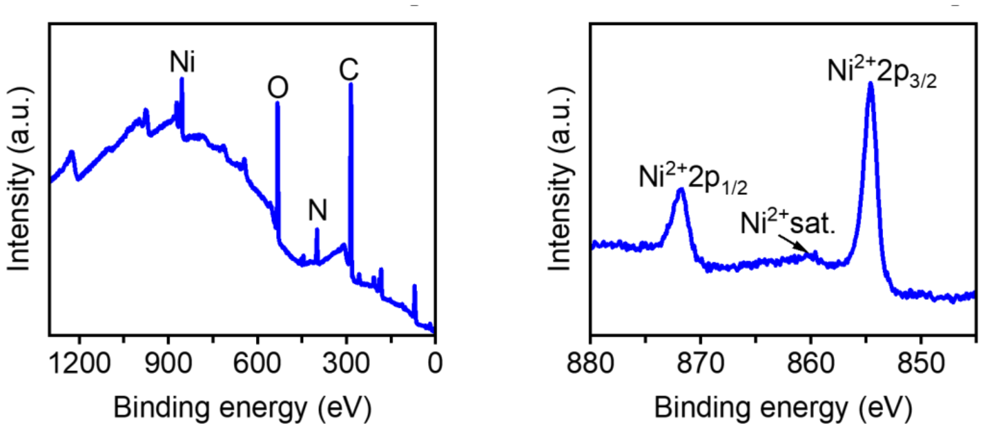

Figure S9. The XPS spectra of [**NiL**]$_n$ metallopolymer film.

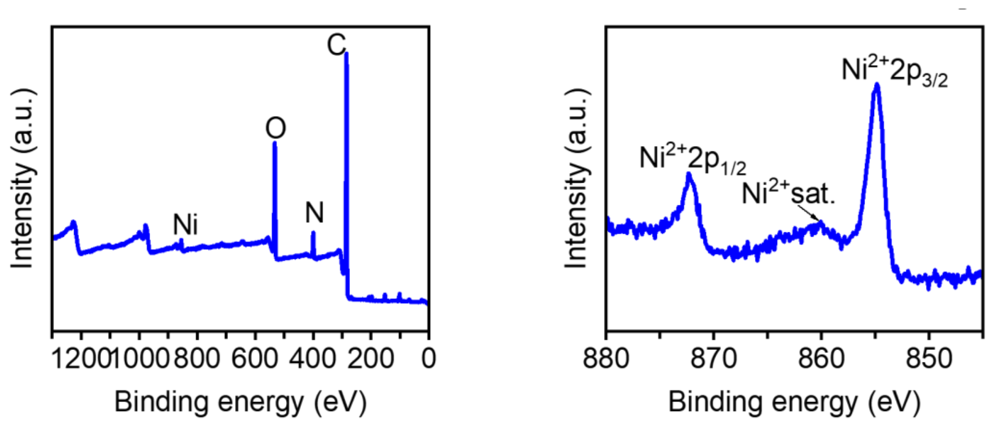

Figure S10. The XPS spectra of [**NiL']ₙ** metallopolymer film.

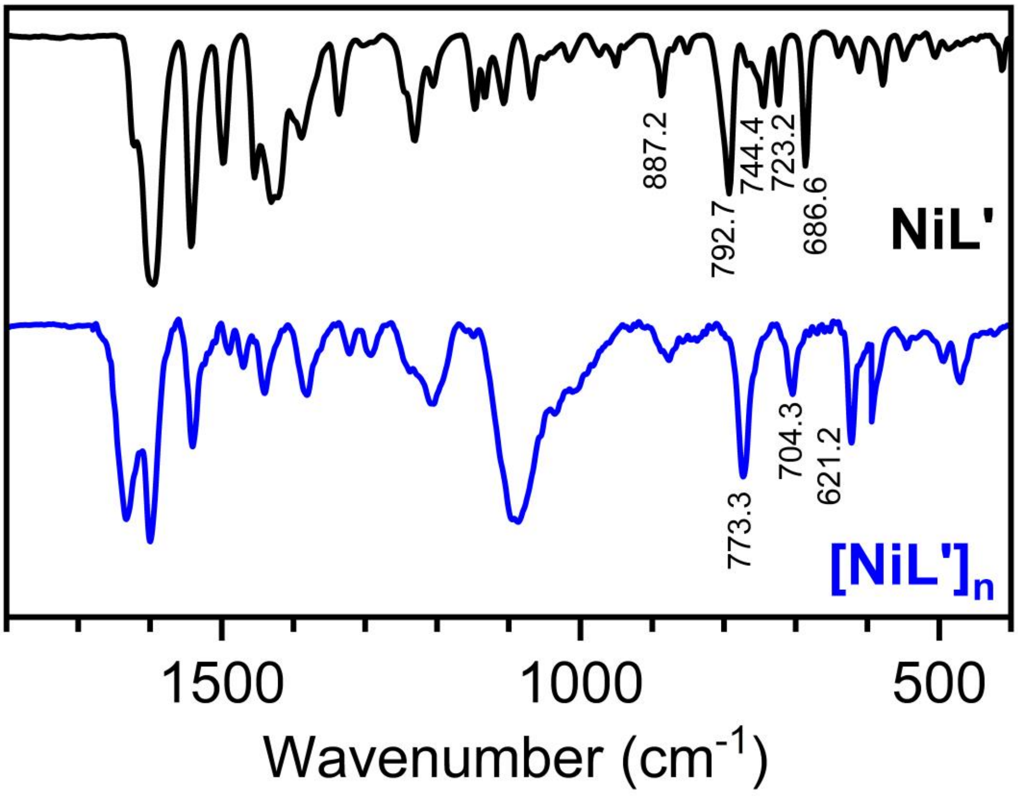

Figure S11. The FT-IR spectra of **NiL'** monomer and [**NiL']$_n$** metallopolymer film.

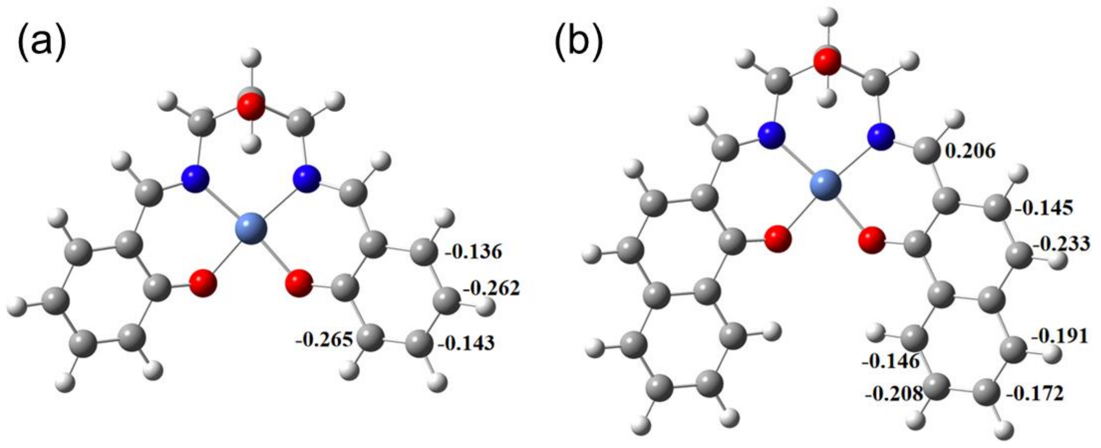

Figure S12. The natural population analysis (NPA) charge of **NiL** and **NiL'**.

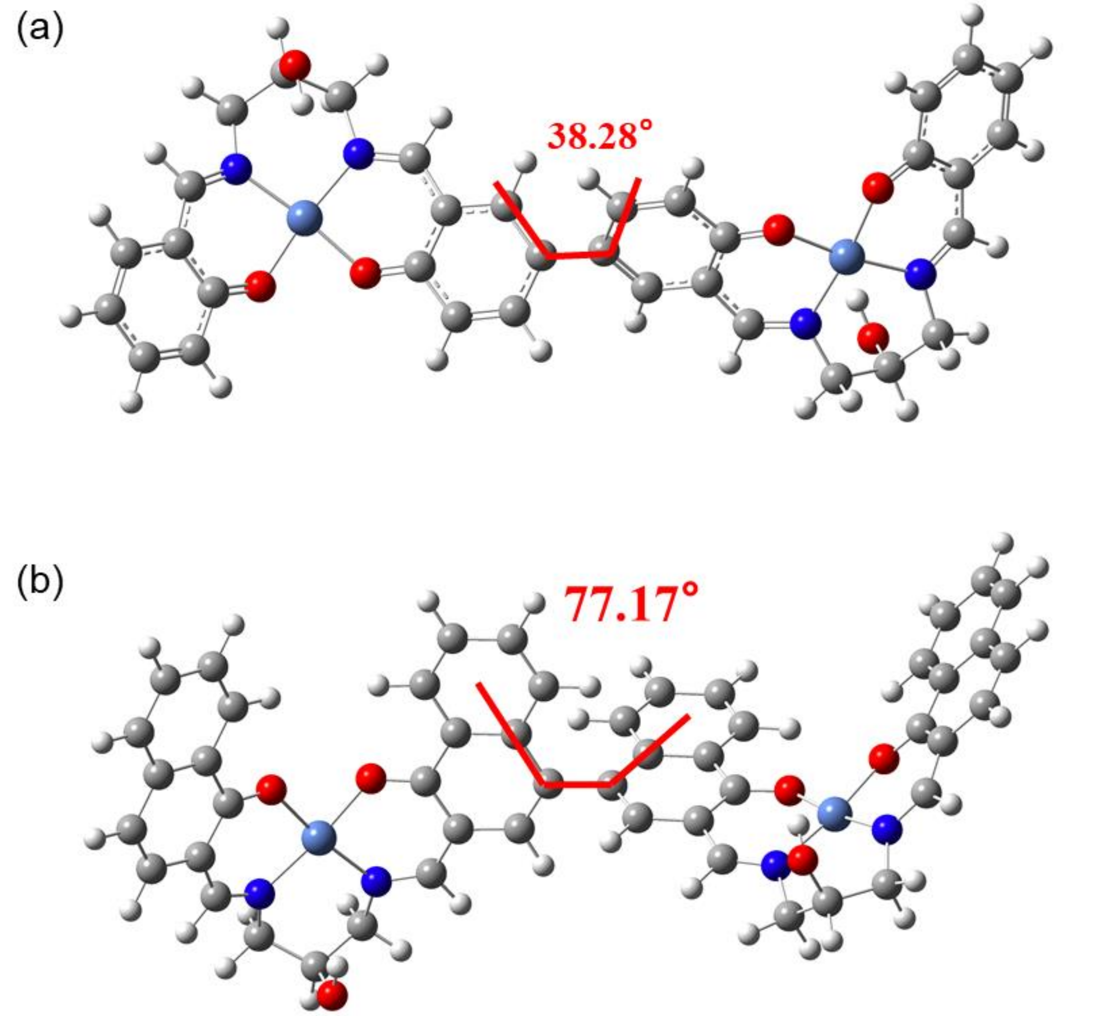

Figure S13. The optimized structure of the dimer (a) **[NiL]$_2$**, and (b) **[NiL']$_2$**, indicating a smaller angle between the two benzene rings in **[NiL]$_2$**.

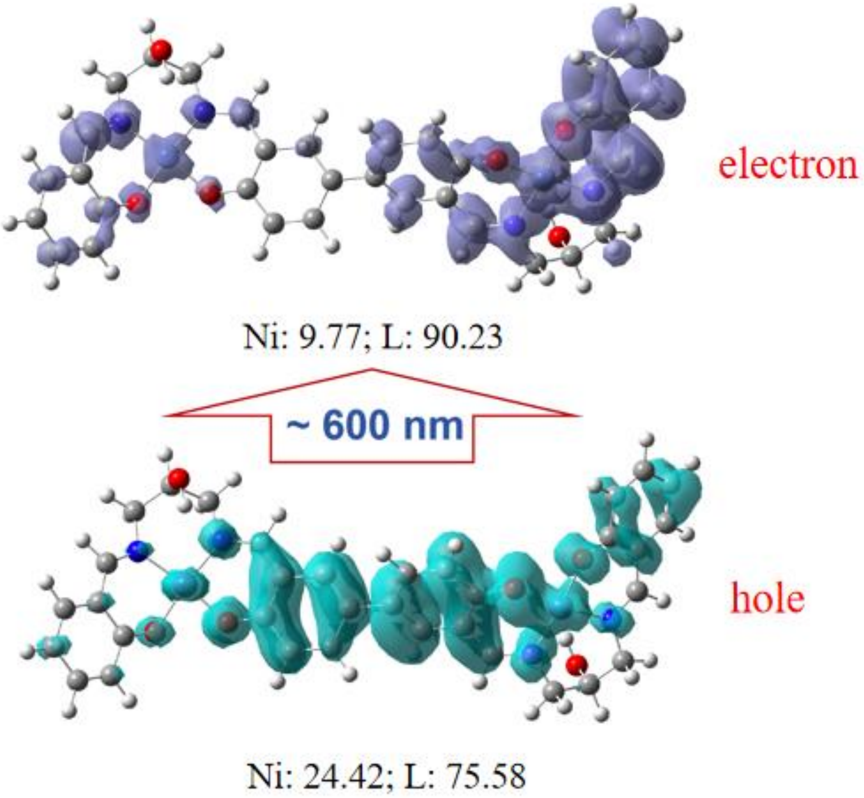

Figure S14. Contour plots of hole and electron (isovalue = 0.0004) in the transition process of ~600 nm and the contribution of molecular fragment (%) for **[NiL]₂**.

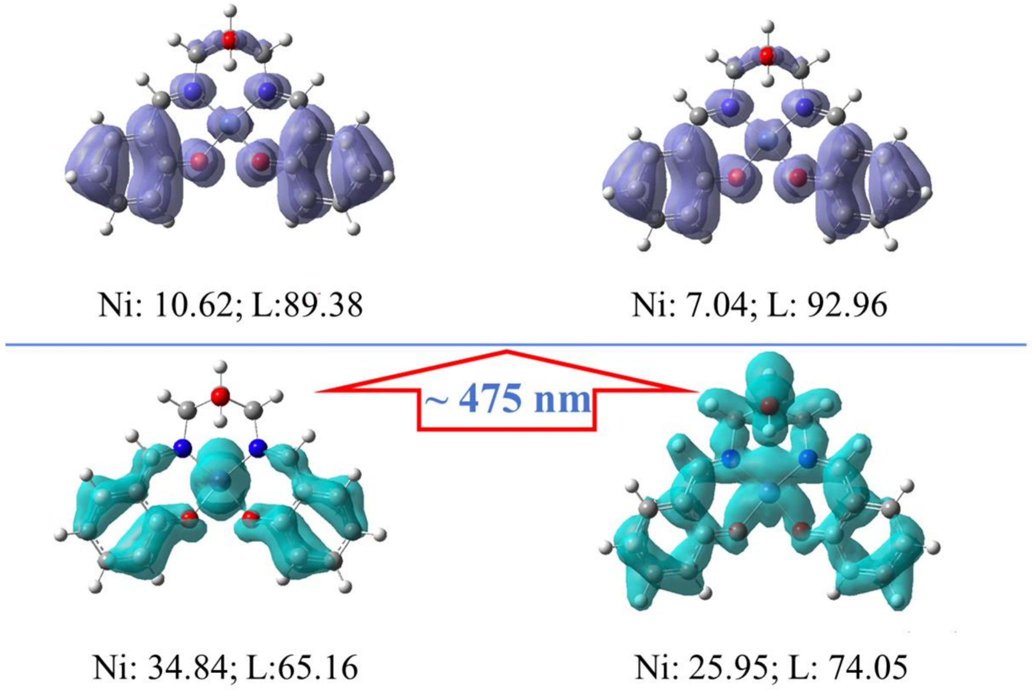

Figure S15. Contour plots of hole and electron (isovalue = 0.0004) in the transition process of 475 nm and the contribution of molecular fragment (%) for **$[NiL]^{2+}$**.

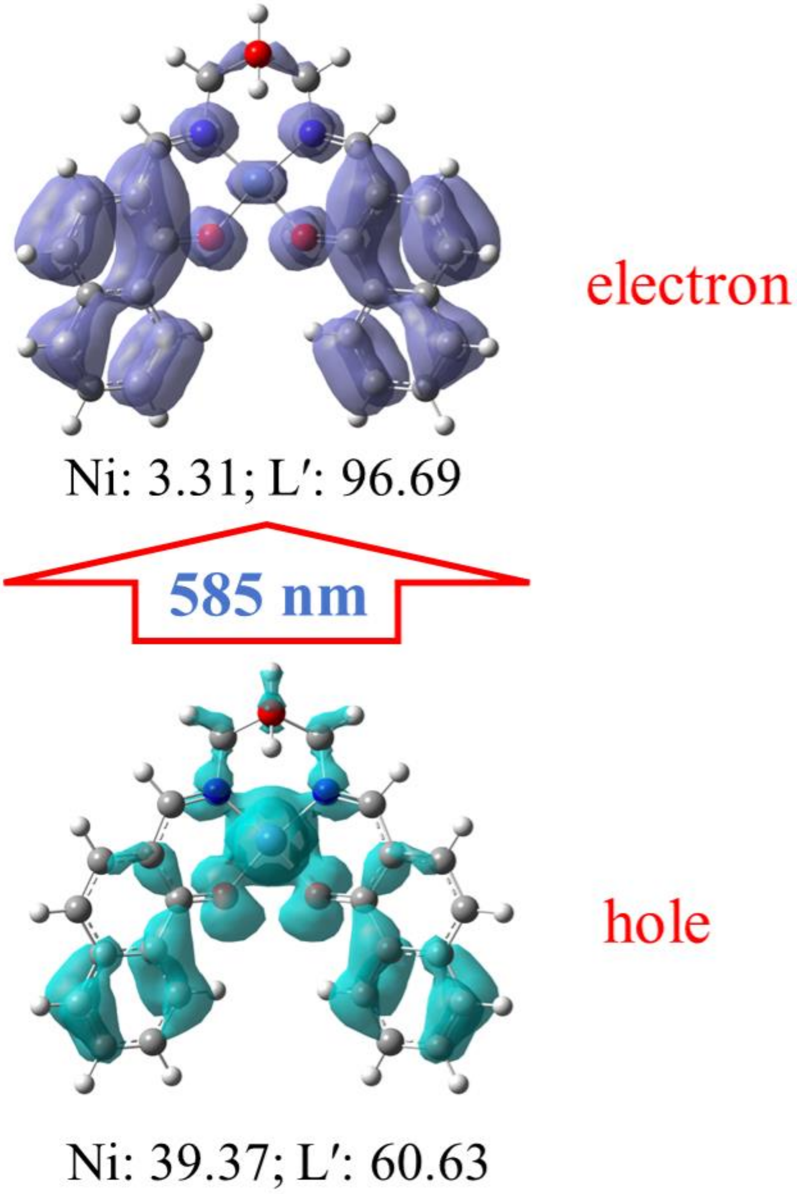

Figure S16. Contour plots of hole and electron (isovalue = 0.0004) in the transition process of 585 nm and the contribution of molecular fragment (%) for **[NiL']$^{2+}$**.

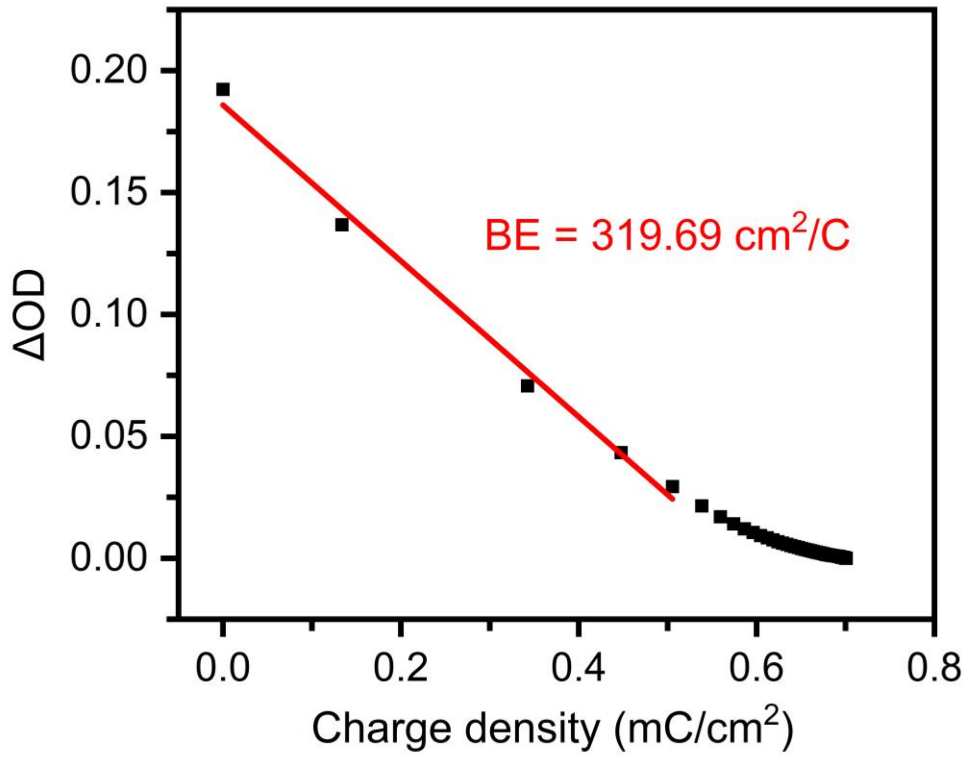

Figure S17. Plot of the optical density (ΔOD) at 475 nm versus the charge density for the **[NiL]ₙ**/TiO_2 electrochromic device during the bleaching process (after applying a potential of 2.1 V) under an applied potential of −0.5 V.

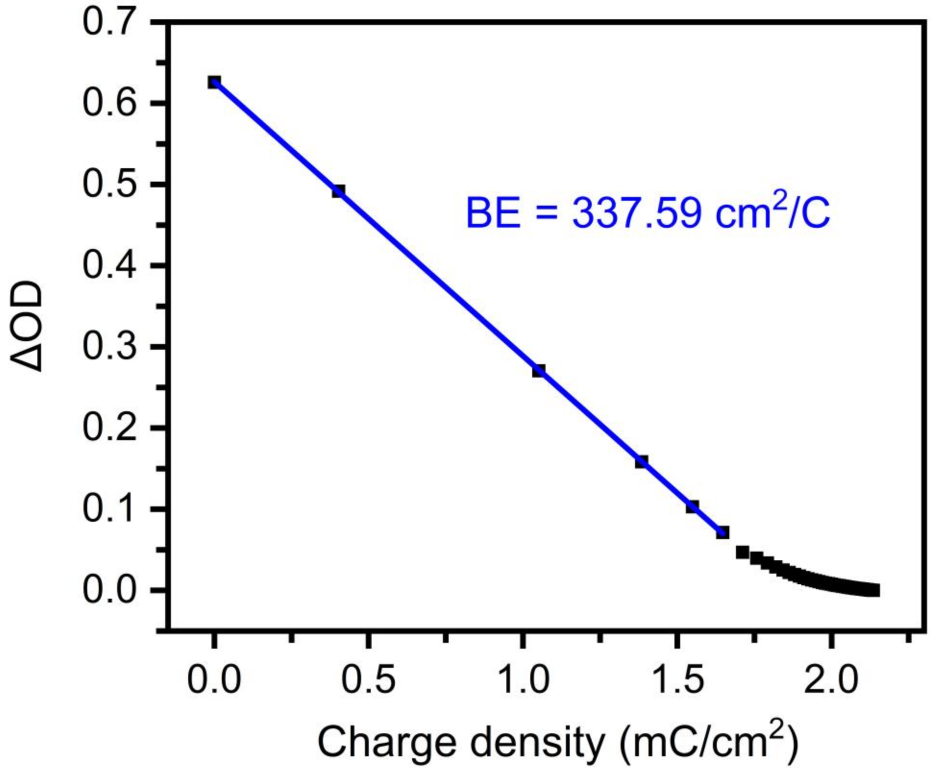

Figure S18. Plot of the optical density (ΔOD) at 585 nm versus the charge density for the **[NiL']n**/TiO_2 electrochromic device during the bleaching process (after applying a potential of 2.1 V) under an applied potential of −0.5 V.
